# What does the COVID-19 pandemic mean for HIV, tuberculosis, and malaria control?

**DOI:** 10.1186/s41182-020-00219-6

**Published:** 2020-05-13

**Authors:** Floriano Amimo, Ben Lambert, Anthony Magit

**Affiliations:** 1 Department of Global Health Policy, Graduate School of Medicine, The University of Tokyo, Tokyo, Japan; 2 Faculty of Medicine, Eduardo Mondlane University, Maputo, Mozambique; 3 MRC Centre for Global Infectious Disease Analysis, School of Public Health, Imperial College London, London, UK; 4 Human Research Protection Program, University of California San Diego School of Medicine, San Diego, CA USA

**Keywords:** COVID-19, HIV, Tuberculosis, Malaria, Health systems, Africa

## Abstract

Despite its current relatively low global share of cases and deaths in Africa compared to other regions, coronavirus disease 2019 (COVID-19) has the potential to trigger other larger crises in the region. This is due to the vulnerability of health and economic systems, coupled with the high burden of human immunodeficiency virus (HIV), tuberculosis (TB), and malaria. Here we examine the potential implications of COVID-19 on the control of these major epidemic diseases in Africa. We use current evidence on disease burden of HIV, TB, and malaria, and epidemic dynamics of COVID-19 in Africa, retrieved from the literature. Our analysis shows that the current measures to control COVID-19 neglect important and complex context-specific epidemiological, social, and economic realities in Africa. There is a similarity of clinical features of TB and malaria, with those used to track COVID-19 cases. This coupled with institutional mistrust and misinformation might result in many patients with clinical features similar to those of COVID-19 being hesitant to voluntarily seek care in a formal health facility. Furthermore, most people in productive age in Africa work in the informal sector, and most of those in the formal sector are underemployed. With the current measures to control COVID-19, these populations might face unprecedented difficulties to access essential services, mainly due to reduced ability of patients to support direct and indirect medical costs, and unavailability of transportation means to reach health facilities. Therefore, if not accompanied with appropriate economic and epidemiological considerations, we anticipate that these measures might result in unprecedented difficulties among vulnerable segments of society to access essential services, including antiretroviral and prophylactic drugs among people living with HIV and Acquired Immune Deficiency Syndrome, anti-tuberculosis drugs, and curative and preventive treatments for malaria among pregnant women and children. This might increase the propensity of patients taking substandard doses and/or medicines, which has the potential to compromise drug efficacy, and worsen health inequalities in the region. COVID-19 responses at country level should include measures to protect vulnerable and under-served segments of society.

## Introduction

As health professionals, communities, governments, and global institutions work closely to halt the spread of coronavirus disease 2019 (COVID-19) and mitigate its societal impact, the number of cases and deaths continues to rise globally. To date, the disease —first reported in Wuhan, China, on 8 December 2019 and declared a pandemic by the World Health Organization (WHO) on 11 March 2020—has been reported in about 185 countries and territories [1]. Of these, 45 are in the WHO African region where human immunodeficiency virus (HIV), tuberculosis (TB), and malaria are endemic. With about 8767 confirmed cases and 413 attributable deaths as of 13 April 2020, the global share of COVID-19 cases and deaths in Africa remains below that of other WHO regions [1, 2].

To date, most analyses have explored the potential effects of the pandemic on the capability of health systems across the continent to deliver essential services, focusing on the response to COVID-19 [3–5]. However, the tremendous risk of COVID-19 on the continent goes beyond shortages of resources and infrastructure needed to directly manage the pandemic. In this study, we focus on the effect of the pandemic on the capacity of patients to use essential health services. We first describe the current dynamics of the pandemic across the WHO African region, using laboratory-confirmed cases and attributable deaths counts from the Center for Systems Science and Engineering at Johns Hopkins University [2]. Subsequently, we examine the potential implications of COVID-19 on other epidemic diseases so far responsible for the largest mortality, morbidity, and disability share on the continent. We use the current evidence on the disease burden of HIV, TB, and malaria, and the epidemic patterns of COVID-19 in the WHO African region retrieved from the literature to support our analysis. The data used in this analysis are drawn from the references provided.

## Analysis and discussion

In the WHO African region, the first COVID-19 case was reported in Algeria on 25 February 2020. Among African Union member states, this was the second case, after the disease was first reported in Egypt on 14 February 2020. Since then, the disease has spread exponentially across the continent. Currently, the region representing approximately 13.7% of the global population (1,019,922,000/7,430,261,000) [6], accounts for about 0.5% (8767 /1,776,157) of the global share of COVID-19 cases and about 0.4% of the global share of attributable deaths (413/108,804) [1, 2] as of 13 April 2020. South Africa has emerged as the epicenter of the disease in the region, accounting for the largest share of cases reported to date [1]. There is a notable cross-country variability in case fatality rate (CFR) in the WHO Africa region [2]. Even so, current data suggest that most countries in the region have death rates that are below the global time delay adjusted CFR estimate of 5.7% (95% confidence interval: 5.5–5.9%) [7].

The relatively low global share of cases and deaths in Africa might be explained in part by the population age structure. This is because clinical severity of COVID-19 increases with age [8], and the populations across the continent are largely young, with a median age of approximately 18.7 years, compared to the global median age of approximately 30.2 years [6]. However, limited availability of detailed patient-level data, under-ascertainment of mild cases, and mis-assignment of causes of death pose important challenges for the accurate estimation of CFR [9]. This means that, in addition to population age structure, the current CFR in the region might also be a reflection of the fact that patients diagnosed with COVID-19 in most African countries are usually those with symptoms as asymptomatic patients might not be easily accessible to the health system. Therefore, a significant portion of COVID-19 cases and attributable deaths across the continent is probably not being accounted for in official statistics. Lack of testing and contact tracing capability is a significant contributor as well.

Despite the relatively low cases counts and death rates in most African countries, COVID-19 poses an unprecedented public health risk on the continent. This is, among other factors [3–5], because of the high burden of other endemic diseases. Globally, the regions with the largest share of people living with HIV/AIDS (PLWHA) are Eastern and Southern Africa, with 20.6 million (18.2–23.2 million), and Western and Central Africa, with 5.0 million (4.0–6.3 million) [10]. Among PLWHA, TB is the leading cause of death. In contrast to COVID-19, CFR of TB is 15.7% in the general population, 32.6% among PLWHA, and 41.0% among patients with Rifampicin-resistant or multidrug-resistant TB [11]. Clinically, TB presents with fever, cough, and dyspnea, among other symptoms. Despite the differences in the duration of incubation and others, these three symptoms are also the clinical features observed in most patients with COVID-19, with prevalence of 88.7% (84.5–92.9%), 57.6% (40.8–74.4%), and 45.6% (10.9–80.4%), respectively [12]. Additionally, the WHO African region accounts for 93% of total malaria cases and 94% of total malaria deaths [13]. There have been important reductions in malaria deaths in the past two decades. To ensure continued reductions in malaria death rates, cases need to be correctly diagnosed and promptly treated. The main diagnostic characteristic of malaria is fever [14, 15]. Therefore, the similarity of symptoms poses important challenges to the current measures to control, not only COVID-19, but TB and malaria as well.

Furthermore, the current measures to control COVID-19 include quarantine of suspected cases, isolation of infected patients, contact tracing, and among other strategies [16, 17]. These might not have good acceptability in some communities [18, 19]. Therefore, many patients with clinical features similar to those of severe acute respiratory syndrome coronavirus 2 (SARS-CoV-2) infection, which might be COVID-19, and/or any other disease with similar symptomatology, might be hesitant to voluntarily seek testing and treatment in a formal health facility. Additionally, in most cases SARS-CoV-2 infection presents itself with mild symptoms. This coupled with fear of high scrutiny that might be associated with a COVID-19 diagnosis may also lead to individuals not seeking care and therefore facilitate the spread of the pandemic. Even though contact tracing is vital to halt transmission, some patients might have privacy concerns [20]. This was observed during the Ebola outbreak in Central and West Africa, in part due to institutional mistrust and misinformation [21]. This might result in an increased household transmission of COVID-19 as infected family members will remain in contact with uninfected patients without any protective measure. Transmission of COVID-19 occurs even among asymptomatic and paucisymptomatic patients. These factors might result in an increased risk of complications and mortality among vulnerable populations because they might delay or avoid seeking care in a formal health facility. For pregnant women with untreated malaria, the complications—usually underestimated—are twofold, comprising maternal and child adverse outcomes [22].

Reduced ability of patients to access health services among under-served and vulnerable segments of society is another critical challenge. To curb the spread of the pandemic, governments are implementing measures, which in addition to limitations to travel and business, also include closure of schools and universities, as well as recommendations for people to stay home. However, most people in productive age in Africa work in the informal sector, and most of those in the formal sector are underemployed [23, 24]. Therefore, if these measures are enforced without appropriate economic and epidemiological considerations, millions of vulnerable populations across the continent might face unprecedented difficulties to access health services due to limited disposable income to support transportation expenses, as well as other direct and indirect medical costs. Among others, these populations include: (1) PLWHA who need to access health facilities regularly to retrieve antiretroviral and prophylactic drugs; (2) patients under treatment for TB who need to go to health facilities regularly to access antituberculosis drugs; (3) approximately 11 million pregnant women exposed to *Plasmodium falciparum* infection annually who need intermittent preventive treatment for malaria prevention as part of antenatal care services; and (4) approximately 24 million children infected with *Plasmodium falciparum* annually who need correct diagnosis and prompt treatment of malaria [13]. This is compounded by the fact that nearly 287,282,013 (29.0%) people and 64,495,526 (28.2%) women of reproductive age need to travel more than 2h to reach the nearest health facility [25]. Additionally, COVID-19 control measures in some countries include limitation of number of passengers in public transportation. This might worsen the availability of transportation means to reach health facilities.

## Conclusions

The current measures to control the pandemic, as they are being enforced across the continent, neglect important and complex context-specific realities in Africa. We anticipate an unprecedented reduction in the capacity of patients to use health services. Limited access to essential medicines is known to create the conditions for patients to use substandard drugs and/or doses. This, in addition to reducing patient outcomes by increasing morbi-mortality due to HIV/AIDS, TB, and malaria, might contribute to the emergence and spread of drug-resistant pathogens. Moreover, reduced access to effective antituberculosis treatment might increase infectiousness of TB patients. This has the potential to trigger other larger crises in the region and could worsen health inequalities and result in reversal of global health gains in key indicators. Therefore, COVID-19 responses at country level should be tailored to local social, epidemiological, and economic profiles. These should include measures to protect under-served and vulnerable populations, in particular PLWHA, patients with co-morbidities and other risk factors, communities living in malaria endemic settings, pregnant women, people under treatment for tuberculosis, and other relevant segments of society. These populations need protection not only from the pandemic, but also from the consequences of its control measures.

## Data Availability

The data used in this analysis are drawn from the references provided.

## Abbreviations

AIDS: Acquired Immune Deficiency Syndrome
CFR: Case fatality rate
COVID-19: Coronavirus disease 2019
HIV: Human immunodeficiency virus
PLWHA: People living with HIV/AIDS
SARS-CoV-2: Severe acute respiratory syndrome coronavirus 2
TB: Tuberculosis
WHO: World Health Organization
